# Genetic mapping of QTL for maize leaf width combining RIL and IF_2_ populations

**DOI:** 10.1371/journal.pone.0189441

**Published:** 2017-12-12

**Authors:** Ruixiang Liu, Qingchang Meng, Fei Zheng, Lingjie Kong, Jianhua Yuan, Thomas Lübberstedt

**Affiliations:** 1 Institute of Food Crops, Provincial Key Laboratory of Agrobiology, Jiangsu Academy of Agricultural Sciences, Nanjing, Jiangsu Province, China; 2 Department of Agronomy, Iowa State University, Ames, Iowa State, United States of America; Washington State Univeristy, UNITED STATES

## Abstract

Leaf width is an important component of plant architecture that affects light capture during photosynthesis and wind circulation under dense planting conditions. To improve understanding of the genetic mechanisms involved in leaf width at different positions, a comprehensive evaluation using the RIL (Recombinant Inbred Line) and IF_2_ (Immortalized F_2_) populations and a subsequent meta-analysis were performed. Forty-seven QTL associated with leaf width at different positions below the tassel were detected. The individual effects of QTL explained 3.5% to 17.0% of the observed phenotypic variation, and ten QTL explained over 10%. The initial QTL were integrated into eight mQTL (meta-QTL) through a meta-analysis. Our results suggested that leaf widths at different positions may be affected by several of the same mQTL and may also be regulated by many different mQTL. These results provide useful information for breeding high density tolerant inbred lines and hybrid cultivars, as well as for using marker-assisted selection for important mQTL.

## Introduction

Over the past several decades, improvements in plant architecture greatly increased maize grain yields [1, 2]. All of leaf size and shape morphological traitsplay important role in determining plant architecture. Leaf size, determinated by leaf width, leaf lengthand leaf area, is an important component of leaf morphology and significantly influences the canopy morphology, photosynthetic activity, and hence grain yield [3]. Leaf width is an important component of leaf size. Smaller and narrower leaf widths decrease shade effects on lower leaves and enhance light capture for photosynthesis in dense plantings with a high leaf area index [4]. Excessive leaf size may decrease grain yield, owing to a decrease in photosynthetically active radiation utilization [5]. Therefore, understanding the genetic mechanisms of maize leaf width at different position would not only address the radiation use efficiency in plant science but also facilitate the breeding of tolerant to high planting density maize with an optimized leaf width.

Leaf development is initiated from the shoot apical meristem (SAM) [6]; then the leaf polarity is established by three main axes, named proximal-distal (longitudinal), midvein-margin (mediolateral) and adaxial-abaxial (dorsoventral); finally the leaf shape and size is determined by a coordinated regulation of cell differentiation and expansion along these axes[7]. Leaf width is determined via differentiation along the mediolateral of founder cells in the peripheral zone of the shoot apical meristem [4]. In maize, several key genes that affect the development of the axes have been identified by using mutants, such as the narrow sheath *ns1* and *ns2* mutants [8], the narrow and threadlike leaf phenotypes of *lbl1* [9] and *rgd2* [10]. These mutants have helped to elucidate the molecular mechanisms of leaf-width development in maize.

In the past several decades, natural variation in maize leaf width has been determined by using quantitative trait locus (QTL) mapping [3, 4, 11–14]. For example, Guo et al. [4] have identified 46 QTL associated with the width of the four consecutive leaves above the uppermost ear in four RIL populations and in three environments. In addition, Yang et al. [3] have detected 83 QTL associated with the width of eight leaves below the tassel. A genome-wide association study (GWAS) method has been used to detect variants at candidate loci and genes responsible for leaf width. Tian et al. [15] have identified 34 QTL for leaf width through NAM-GWAS (nested association mapping population). Additionally, Yang et al. [16] have found 18 SNPs associated with ear leaf width. Current research is often focus on the leaves (one to three) near the ear, owing to their effect on grain yield; only two QTL mapping studies on leaves at different positions have been published to date [3, 4], and these studies have shown inconsistent results regarding the QTL regions. This discrepancy may limit the application of these QTL in marker-assisted selection. Therefore, further investigation of the genetic basis of leaf width at different positions and the heterosis for leaf width is needed.

In this study, we conducted a comprehensive genetic dissection of leaf width to assess the genetic architecture of seven consecutive leaves, by using a RIL and IF_2_ population derived from a cross between the Chinese elite inbred lines S951 and Qi319. The major objective of this study was to improve understanding of the genetic basis (including additive and dominance effect) of leaf width at different positions and to determine how leaf width affects plant architecture and adaptation to high density planting. In addition, we sought to identify and fine map major leaf width QTL.

## Materials and methods

### Plant materials and leaf width collection

A total of 164 F_9_ RIL families derived from a cross between inbreds S-951 and Q319 was used for leaf width analysis. The parents of this population were chosen on the basis of distinct maize germplasm groups. S-951 is an inbred line derived from Chinese Stiff Stalk germplasm, a heterotic group widely used in China, whereas Qi319 is an inbred line derived from Chinese non-Stiff Stalk germplasm, also widely used in China. Similar to the procedure for generating the previously described intercrossed F_2_ population [17, 18], the 164 RILs were randomly divided into two groups of 82 RILs. Single crosses were randomly conducted between the two groups. Each RIL was used only once in each group of matings to generate crosses. This procedure was repeated four times to produce 328 single crosses, forming the IF_2_ population. The RIL and IF_2_ populations were planted in Dafeng, Liuhe in 2013, and Sanya in 2014. At three year-location combinations, populations of RIL and IF_2_ were in neighboring blocks, each planted in a randomized complete block design with three replications. Each plot was singlerow, with 4 m long and 0.67 m between rows. The population density was 45,000 plants per ha. Ten days after pollen shedding, five consecutive plants from the middle of each plot were chosen to evaluate the top seven leaves’ width (LW). The seven consecutive leaves below the tassel were designated leaves one to seven and named L1W, L2W, L3W, L4W, L5W, L6W and L7W, respectively. LW and trait value was determined according to the methods described by Guo et al. [4].

### Molecular markers and genetic linkage maps

The 164 RILs were genotyped with 209 polymorphic SSR markers that covered the whole genome. The genotypes of each hybrid in the IF_2_ population were deduced from the genotype of both its RIL parents. A linkage map was constructed by using the SSR genotypes of the 164 RILs. Molecular linkage maps for the RILs and IF_2_ population were constructed by using ICIMapping [19] with a logarithm-of-odds (LOD) threshold of 3.0. The Kosambi mapping function was used for calculating map distances [20].

### Data analysis and QTL Mapping

The broad-sense heritability (*H*^2^) for each leaf width below the tassel was calculated according to the method described by Guo et al. [21], as the genetic variance (*V*_G_) divided by the sum of genetic variance (*V*_G_), *G* × *E* variance (*V*_G×E_) and error variance (*V*_ε_), based on the plot basis. The calculation of best linear unbiased predictors (BLUPs) for each line in RILs and each hybrid in IF_2_ was done with a mixed linear model that accounted for the effects of environment, replication, genotype, and genotype by environment described by Guo et al. [21]. The estimated genetic variance and error variance by analysis of variance were for BLUP estimators. The BLUPs for each line and hybrid from three locations were used in QTL mapping [21].

The mapping of QTL were performed with QTL ICIMapping software (http://www.isbreeding.net) [22]. QTL were identified using the ICIM-ADD mapping method with the default software parameters (PIN (probability in stepwise regression) = 0.001, step = 1.0 cM). The threshold levels for declaring the existence of a QTL with an additive and/or dominance effect were determined by performing 1,000 permutations on the data with a significance level of P≤0.05. Gene action was determined by the ratio of the absolute value of the estimated dominance effect divided by the absolute value of the estimated additive effect following [23] (additive 0–0.20, partial dominance 0.21–0.80, dominance 0.81–1.20, over dominance>1.20).

### QTL integration and meta-analysis

The meta-analysis method was used to integrate the QTL information identified in the RIL and IF_2_ population, and performed using BioMercator 4.2 [24, 25]. The significant QTL model for indicating the number of meta-QTL(mQTL) in each chromosome was determined by the lowest Akaike information criterion (AIC) values as described by [26]. The number of mQTL that showed the best fit to the results in a given linkage group was determined on the basis of a modified Akaike criterion [27]. The names of the mQTL were assigned according the following nomenclature: "m" + "QLW" + "chromosome number" + "meta-QTL number" described by Guo et al. [4].

## Results

### Trait performance

The inbred line Qi319 showed decreased values for consecutive leaf-widths below the tassel, as compared with those of the inbred line S-951. The values of L1W, L5W, L6W, and L7W were markedly lower than those of S-951, with the exception of L2W, L3W, and L4W, which presented no significant differences (Table 1). Among IF_2_ families, trait values were generally higher than those of RILs. For most traits, phenotypic values were normally distributed, with increased variation in both the RIL and IF_2_ populations. All leaf widths exhibited substantial bidirectional transgressive segregation, thus supporting a polygenic quantitative genetic control of these traits. Broad-sense heritabilities ($h_{b}^{2}$) for each leaf width ranged from 0.61 (L1W) to 0.78 (LW4) in the RIL population and from 0.62 (L1W) to 0.80 (L4W) in the IF_2_ population, thus indicating that genetic factors are determinants of these traits.

**Table 1:** Leaf widths in parental lines, RIL, and IF_2_ populations across three environments.

Trait		L1W	L2W	L3W	L4W	L5W	L6W	L7W
**S951**	Mean±SE	5.13±0.13	8.07±0.10	9.27±0.15	10.52±0.13	11.01±0.15	10.73±0.09	9.27±0.09
**Qi319**	Mean±SE	6.41±0.14**	8.24±0.08	9.12±0.12	9.85±0.11	9.05±0.11**	8.11±0.12**	5.85±0.07**
**IF2**	Mean±SE	4.81±0.33	6.69±0.52	8.23±0.58	9.22±0.62	9.59±0.56	9.34±0.51	8.79±0.49
	Range	3.97–7.89	5.36–9.41	6.57–10.79	7.70–11.41	8.21–11.59	8.24–11.87	7.48–10.67
	Skewness	0.28	0.234	-0.10	-0.054	-0.13	0.04	0.13
	Kurtosis	0.28	0.51	0.13	0.12	0.34	0.10	-0.04
	$h_{b}^{2}$	0.62	0.76	0.78	0.80	0.77	0.70	0.68
**RIL**	Mean±SE	4.79±0.38	6.52±0.59	7.83±0.62	8.54±0.63	8.66±0.54	8.02±0.58	7.10±0.56
	Range	3.84–6.82	5.23–8.81	6.35–9.56	6.72–10.95	6.88–11.52	6.41–11.02	5.69–9.81
	Skewness	0.20	0.26	0.13	0.09	-0.24	0.05	0.33
	Kurtosis	-0.08	0.02	0.19	0.28	0.06	-0.11	-0.35
	$h_{b}^{2}$	0.61	0.74	0.76	0.78	0.73	0.68	0.67

Note: $h_{b}^{2}$ broad-sense heritability

** significant at 0.01 level of probability by using Student's t-test.

Phenotypic correlation coefficients among LW traits (Table 2) ranged from 0.22 to 0.90 in the RIL and 0.50 to 0.92 in the IF_2_ population. Phenotypic correlations were significant and positive, and closer phenotypic correlations existed between adjacent leaves than non-adjacent leaves, and decreased with the distance of leaves [3, 4].

**Table 2:** Phenotypic correlations among traits in the RIL population (below diagonal) and the IF_2_ population (above diagonal).

	L1W	L2W	L3W	L4W	L5W	L6W	L7W
**L1W**		0.86**	0.81**	0.71**	0.65**	0.56**	0.50**
**L2W**	0.89**		0.91**	0.85**	0.75**	0.65**	0.56**
**L3W**	0.77**	0.90**		0.91**	0.83**	0.73**	0.64**
**L4W**	0.71**	0.84**	0.90**		0.92**	0.82**	0.74**
**L5W**	0.52**	0.67**	0.75**	0.89**		0.92**	0.86**
**L6W**	0.37**	0.49**	0.58**	0.73**	0.86**		0.92**
**L7W**	0.22**	0.33**	0.43**	0.57**	0.71**	0.89**	

** significant at 0.01 level of probability by using Student's t-test.

### Identification of major leaf width QTL in the RIL

Threshold values (P<0.05 significance level) were determined with 1000 permutations of the leaf width data. In the RIL population, a total of 17 QTL were identified for the seven leaf widths across chromosomes 1, 2, 5, 7, 8 and 9 (Table 3). The phenotypic variance of individual QTL ranged from 6.5% (contributed by qL2WR1) to 17.0% (qL3WR5), with seven QTL explaining more than 10% of the phenotypic variation. Among these QTL, three were associated with L1W, three with L2W, two with L3W, two with L4W, two with L5W, three with L6W and two with L7W. Fifteen positive alleles among the 17 QTL originated from S-951 and contributed to increased leaf width values. The QTL qL2WR5, qL3WR5 and qL4WR5 were detected within the marker interval umc1019-bnlg118 on chromosome 5 and explained more than 10% of the total phenotypic variation. The QTL qL2WR7 and qL3WR7 were detected within the marker interval mmc0411-umc1015 on chromosome 7 and explained 8.9% and 10.9% of the observed phenotypic variation, whereas QTL qL4WR7 and qL5WR7 were identified near the same marker interval phi008-mmc041 and explained 7.0% and 8.6% phenotypic variation, respectively. The QTL qL1WR2 was detected within the marker interval bnlg125-umc2248 and accounted for 9.8% of the total phenotypic variation, with a decreasing leaf width additive effect of 0.15. The QTL qL5WR5 was located on chromosome 5 between umc1680 and umc1019 and accounted for 13.4% of the total phenotypic variation.

**Table 3:** QTL detected in the RIL population.

TraitName^a^	QTL_name	Chr^b^	Position^c^	LeftMarker	RightMarker	LOD^d^	R^2^(%)^e^	Add^f^
L1W	qL1WR2	2	45	bnlg125	bnlg2248	4.7	9.8	-0.15
L1W	qL1WR7	7	217	umc2334	umc1799	3.8	6.9	0.12
L1W	qL1WR9	9	167	bnlg619	umc1277	3.9	13.5	-0.17
L2W	qL2WR1	1	270	phi265454	umc1681	3.0	6.5	0.16
L2W	qL2WR5	5	217	umc1019	bnlg118	4.6	13.6	0.23
L2W	qL2WR7	7	101	mmc0411	umc1015	3.9	8.9	0.18
L3W	qL3WR5	5	220	umc1019	bnlg118	5.9	17.0	0.29
L3W	qL3WR7	7	101	mmc0411	umc1015	5.4	10.9	0.23
L4W	qL4WR5	5	222	umc1019	bnlg118	5.1	15.8	0.30
L4W	qL4WR7	7	100	phi008	mmc0411	3.6	7.0	0.19
L5W	qL5WR5	5	203	umc1680	umc1019	4.5	13.4	0.21
L5W	qL5WR7	7	100	phi008	mmc0411	3.7	8.6	0.17
L6W	qL6WR2	2	187	umc1551	umc1525	4.3	10.2	0.20
L6W	qL6WR5	5	193	mmc0282	umc1680	3.1	7.9	0.18
L6W	qL6WR8	8	140	bnlg2046	umc1161	3.8	8.4	0.18
L7W	qL7WR2	2	188	umc1551	umc1525	3.1	8.6	0.16
L7W	qL7WR8	8	54	bnlg1176	bnlg1599	3.3	8.9	0.16

Note

^a^Represent the leaves’ width as described in material and methods.

^b^Represent the chromosome number.

^c^The genetc distance (centiMorgans, cM) of the QTL on the relevant chromosome in the genetic linkage map.

^d^The threshold LOD values were determined with 1000-times permutations of the data.

^e^The proportion of phenotypic variation explained by each QTL.

^f^The estimated additive effect of the QTL.

### Identification of major leaf width QTL in the IF_2_

A total of 30 QTL were identified for the seven leaf widths across the whole genome, except for chromosome 4, 6 and 10, in the IF_2_ population (Table 4). Among these QTL, seven were associated with L1W, six with L2W, six with L3W, four with L4W, three with L5W, two with L6W and two with L7W. The phenotypic variance explained by individual QTL ranged from 3.5% (qL2WF7) to 12.8% (qL2WF1.1), whereas four of the QTL accounted for more than 10% of the phenotypic variation (Table 4). The analysis of the positive or negative effects of QTL revealed that 60% (18/30) of the leaf width QTL were associated with an increase in leaf width. The allelic effect distribution of different leaf widths was different. For L1W, 4 of 7 (57.1%) QTL were associated with a decrease in leaf width. However, in contrast to L4W, all QTL tended to increase leaf width. Three QTL, including qL2WF5, qL3WF5 and qL5WF5, were detected on chromosome 5 within the marker interval umc1680 and umc1019 and showed partial dominance. QTL qL1WF5 was detected in the marker interval umc1680-umc1019 on chromosome 5 and had additive effects from parent S-951 and explained 5.6% of the total phenotypic variance. The QTL qL4WF8 on chromosome 8 showed over-dominance and accounted for 5.2% of the phenotypic variance. The QTL qL6WF2.2 was located on chromosome 2, showed dominance, and accounted for 7.9% of phenotypic variance. The three QTL qL1WF7.2, qL3WF7.2 and qL4WF7 explained 4.6%, 6.2% and 5.7% of the phenotypic variance, respectively, and were associated with increased leaf width values, had additive effects, and were detected in the marker interval mmc0411-umc1015 on chromosome 7. The QTL qL1WF1.1, qL2WF1.1 and qL3WF1 accounted for 11.9%, 12.8% and 9.2% of the total phenotypic variation, respectively, and were detected in the region between bnlg1055 and umc1009 on chromosome 1. L1W, L2W and L3W were controlled by one or two large-effect QTL in addition to several small-effect QTL. L4W, L5W, L6W and L7W were controlled by small-effect QTL. These results suggested that the genetic architecture of L1W, L2W and L3W is controlled by one or two large-effect QTL and a few small-effect QTL. The architecture of L4W, L5W, L6W and L7W is complex and is controlled by many small-effect QTL.

**Table 4:** QTL detected in the IF_2_ population.

Trait Name	QTL name	Chr	Position	Left Marker	Right Marker	LOD	R^2^(%)	Add	Dom	Gene action
L1W	qL1WF1.1	1	293	bnlg1055	umc1009	13.1	11.9	0.19	-0.05	PD
L1W	qL1WF1.2	1	325	umc1009	umc1797	5.5	4.1	-0.12	0.03	PD
L1W	qL1WF2	2	16	bnlg1297	mmc0111	8.9	8.2	-0.16	-0.05	PD
L1W	qL1WF3	3	19	phi453121	bnlg1647	6.7	6.7	-0.13	0.06	PD
L1W	qL1WF5	5	211	umc1680	umc1019	6.6	5.6	0.14	0.01	A
L1W	qL1WF7.1	7	6	umc2177	umc1378	5.3	4.8	-0.12	0.00	A
L1W	qL1WF7.2	7	103	mmc0411	umc1015	5.8	4.6	0.12	0.01	A
L2W	qL2WF1.1	1	288	bnlg1055	umc1009	16.8	12.8	0.31	-0.01	A
L2W	qL2WF1.2	1	325	umc1009	umc1797	5.6	3.8	-0.18	0.04	PD
L2W	qL2WF5	5	211	umc1680	umc1019	12.1	10.0	0.31	-0.08	PD
L2W	qL2WF7	7	9	umc2177	umc1378	4.2	3.5	-0.16	0.04	PD
L2W	qL2WF9.1	9	9	bnlg1724	bnlg1583	5.7	4.0	0.19	-0.01	A
L2W	qL2WF9.2	9	50	phi033	phi027	8.0	6.0	-0.23	0.01	A
L3W	qL3WF1	1	288	bnlg1055	umc1009	10.2	9.2	0.26	-0.03	A
L3W	qL3WF5	5	209	umc1680	umc1019	9.7	10.9	0.32	-0.08	PD
L3W	qL3WF7.1	7	11	umc2177	umc1378	4.0	3.7	-0.16	0.07	PD
L3W	qL3WF7.2	7	101	mmc0411	umc1015	6.7	6.2	0.22	-0.01	A
L3W	qL3WF8	8	40	phi115	phi121	4.0	3.8	0.16	0.04	PD
L3W	qL3WF9	9	51	phi033	phi027	4.4	3.8	-0.17	-0.04	PD
L4W	qL4WF1	1	287	umc2241	bnlg1055	8.3	9.0	0.26	-0.01	A
L4W	qL4WF5	5	215	umc1019	bnlg118	7.2	8.5	0.29	-0.12	PD
L4W	qL4WF7	7	101	mmc0411	umc1015	5.3	5.7	0.22	0.00	A
L4W	qL4WF8	8	2	bnlg1194	bnlg1067	4.4	5.2	0.07	0.26	OD
L5W	qL5WF1	1	256	bnlg1268	phi265454	4.9	9.2	0.24	0.01	A
L5W	qL5WF2	2	41	bnlg2277	bnlg125	4.5	6.0	-0.20	0.07	PD
L5W	qL5WF5	5	207	umc1680	umc1019	4.3	7.7	0.25	-0.06	PD
L6W	qL6WF2.1	2	41	bnlg2277	bnlg125	4.5	5.4	-0.18	0.04	PD
L6W	qL6WF2.2	2	132	bnlg1662	bnlg1863	4.5	7.9	0.20	-0.17	D
L7W	qL7WF2.1	2	41	bnlg2277	bnlg125	5.1	5.8	-0.19	-0.02	A
L7W	qL7WF2.2	2	132	bnlg1662	bnlg1863	6.2	9.7	0.23	-0.15	PD

Note: A: additive effect; PD: partial dominance effect; D: dominance effect; OD: overdominance effect

### Comparison of QTL positions between RIL and IF_2_

In the present study, a total of 47 QTL (17 in the RILs and 30 in the IF_2_ population) for the seven leaf-widths were identified by QTL mapping, thus revealing that the IF_2_ population had greater power to detect QTL. To evaluate the genetic overlap among different positions of leaf widths in the RIL and IF_2_ population, the QTL marker intervals were compared. On the basis of the same marker intervals, all QTL were distributed into 25 marker intervals in the maize genome. Non-common marker interval regions controlled all seven leaf-width in different leaf positions, althought the seven leaf-width were correlated highly. This finding was similar to previous results [3]. Of these 25 regions, 14 (56%) were specific to different positions and leaf widths. For example, five marker intervals (bnlg1297-mmc0111 and bnlg125-bnlg2248 on chromosome 2, phi453121-bnlg1647 on chromosome 3, umc2334-umc1799 on chromosome 7 and bnlg619-umc1277 on chromosome 9) affected only L1W; the marker intervals phi115-phi121 on chromosome 8, bnlg1268-phi265454 on chromosome 1 and bnlg1176-bnlg1599 on chromosome 8 were specific to L3W, L5W and L7W, respectively. Eleven (44%) regions exhibited pleiotropic effects on leaf widths at different positions. The umc1680-umc1019 marker interval found on chromosome 5 was responsible for L1W, L2W, L3W and L5W, whereas the mmc0411-umc1015 interval on chromosome 7 was responsible for L1W, L2W, L3W and L4W. There were ten regions that controlled adjacent leaf width, such as the marker interval bnlg1055-umc1009 and umc2177-umc1378, which controlled L1W, L2W and L3W; the phi008-mmc0411 regions, which controlled the L4W and L5W; and the bnlg1662-bnlg1863 and umc1551-umc1525 marker intervals, which were responsible for L6W and L7W. These regions appeared to harbor pleiotropic locus affecting different positional widths of leaves. These results may explain why adjacent leaf widths exhibited a higher phenotypic correlation.

### Meta-QTL analysis

**Fig 1:**
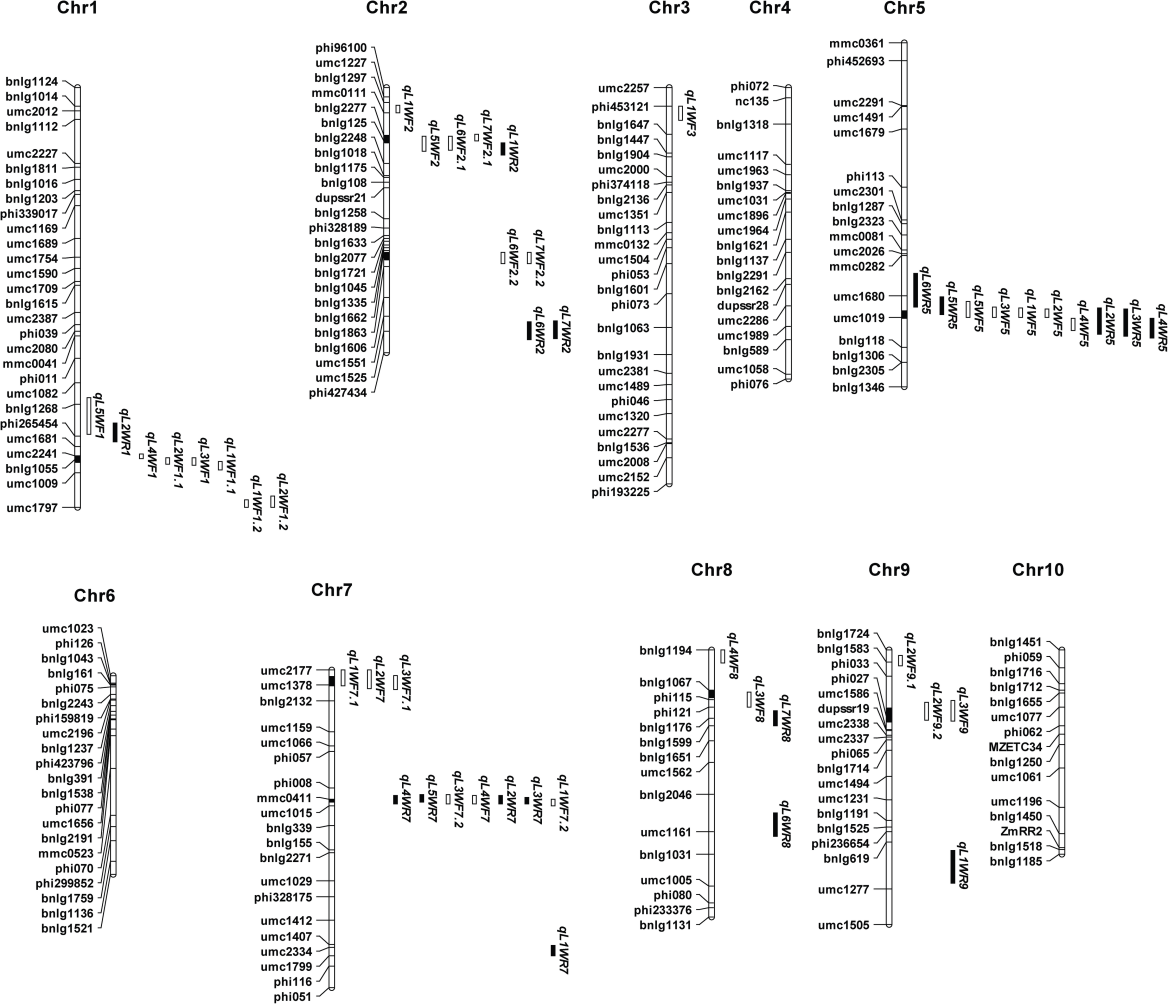
Initial QTL and mQTL detected for the seven leaf widths. The “Chr” represents chromosome. The white bar and the bold bar represents the initial QTL detected in IF_2_ and RIL population respectively. The bold segments in chromosome represent the region of mQTL.

To identify stable and consistent QTL between the RIL and IF_2_ populations, as well as to discriminate between pleiotropic and linked QTL for the seven leaf widths, the initial QTL were analyzed via meta-analysis. Eight mQTL were identified from the 47 initial QTL on the basis of the variation in leaf width (Table 5). The eight mQTL were identified on six chromosomes: one on chromosome 1, 5, 8 and 9, and two on chromosome 2 and 7. On average, one mQTL included 5 initial QTL and ranged from 2 to 10 QTL (Fig 1). Importantly, 10 of the initial QTL that showed an R^2^ > 10% in the RIL and IF_2_ populations were included in 4 of the mQTL, including mQLW1.1, mQLW2.2, mQLW5 and mQLW7.2. mQLW5 included 10 QTL associated with the widths of the first to sixth leaves in the RIL and IF_2_ populations and explained 5.6%-17.0% of the total phenotypic variation. mQLW7.2 comprised seven QTL associated with the widths of the first to fifth leaves in the two populations and explained 4.6%-10.9% of the phenotypic variation. mQLW1 included six QTL associated with the widths of the first to fifth leaves in the two populations and explained 6.5%-12.8% of the phenotypic variation. Fine mapping of these meta-QTL is a reliable strategy for QTL cloning, which is currently underway in our laboratory. Importantly, the initial QTL included in mQLW7.1 was associated with L1W, L2W, and L3W, while the initial QTL included in mQLW2.2 was associated with L6W and L7W.

**Table 5:** Meta-QTL (mQTL) for the seven consecutive leaf-widths below the tassel in the RIL and IF_2_ populations.

QTL cluster	Chr	Left Marker	Right Marker	Original QTL NO.	Pop	Trait	Reference	Candidate gene
*mQLW7*.*1*	7	umc2177	umc1378	3	IF_2_	L1W, L2W, L3W	[11, 15]	
*mQLW9*	9	phi033	phi027	2	IF_2_	L2W, L3W		
*mQLW5*	5	umc1680	umc1019	10	IF_2_, RIL	L1W, L2W, L3W, L4W, L5W, L6W	[3, 16, 28]	*yabby15*[12], *ZmPIN1b*[29]
*mQLW1*	1	umc2241	bnlg1055	6	IF_2_, RIL	L1W, L2W, L3W, L4W, L5W	[4, 11]	
*mQLW7*.*2*	7	mmc0411	umc1015	7	IF_2_, RIL	L1W, L2W, L3W, L4W, L5W		
*mQLW8*	8	bnlg1194	bnlg1067	3	IF_2_, RIL	L3W, L4W, L7W	[3, 15, 30]	
*mQLW2*.*2*	2	bnlg1863	bnlg1606	4	IF_2_, RIL	L6W, L7W	[13]	
*mQLW2*.*1*	2	mmc0111	bnlg2277	5	IF_2_	L1W, L5W, L6W, L7W	[3, 13, 28]	*lg1*[31]; *mrl1*[10]

### Association between leaf-width mQTL and known genes

A mutant gene is a logical candidate gene, if it alters the expression of a target trait and is located within the QTL region associated with that trait [32]. Based on this, we investigated the association between eleven mQTL that were observed in our study and known mutant genes affecting leaf size in maize. The results showed that several mutant genes responsible for leaf size corresponded to the mQTL region (Table 5). The *lg4a* [33] genes had map positions neighboring mQLW8. mQLW2.1 was located close to the *mrl1* gene, which played important role in development of the mediolateral axis [10]. mQLW5 was located near the *ZmPIN1b* gene [29], which mediates auxin transport, increases auxin concentrations in specific tissues and controls gene expression. However, these mutant genes and the meta-QTL might be located too far to provide effective validation. Isolation of the near-isogenic lines with enriched molecular markers (which is underway for some of the mQTL) and fine mapping of these mQTL will help to determine, whether these mutant candidate genes are the causal genetic variants of the mQTL.

## Discussion

### Genetic structure of leaf width

One important maize breeding goal is to increase the stress tolerance for high-density planting of new hybrids, which can be achieved by improving the plant type and canopy architecture [2, 34]. Leaf width is an important trait for high-density planting tolerance in maize breeding. Therefore, unraveling the genetic mechanisms underlying leaf width is essential. Several QTL mapping studies on maize leaf width have been published [3, 4, 12, 18, 28, 35], although they are inconsistent in regard to the QTL regions. Therefore, further investigations addressing the QTL that underlie the phenotypic variance in leaf width, especially in different leaf positions, are required. In this study, we measured the widths of seven consecutive leaves below the tassel, performed phenotypic data analyses, conducted QTL identification, and attempted to investigate the genetic controls underlying leaf width in maize, by using RIL and IF_2_ populations. The results of the phenotypic analysis showed that the width of the seven leaves displayed significantly positive correlations, especially for widths of adjacent leaves. The number of significant QTL for different leaf widths at different positions ranged from 4 (L7W) to 10 (L1W) and the asymmetric and clustered distribution among genomic regions revealed the complex nature of leaf width. Among 30 leaf-width QTL identified in IF_2_ populations, 12 QTL (40%) showed additive gene action, 16 QTL (53.3%) showed partial dominance, and one each showed overdominance and dominance. Collectively, these data indicate that the inheritance of leaf width traits is controlled by a few major QTL and numerous minor QTL. Additive and partial dominance effects play important roles in controlling leaf width.

### Meta-QTL associated with different leaf widths at different positions

The leaf width of adjacent leaves of maize plants showed higher correlations than non-adjacent leaves, thus indicating that the more common QTL can be expected in adjacent leaf widths. As expected, the more common QTL (approximately 70.2% of total loci) were found in leaf widths of adjacent leaves. The findings from several studies are in agreement with this result. Fifty percent of the total loci were observed in adjacent leaf widths in one 253 RIL line derived from a cross between B73 and SICAU1212 [3]. In multiple RIL populations, 48.6, 44.4, 47.5, and 25% of the corresponding QTL were responsible for the widths of adjacent leaves [4]. However, only 6.5% were associated with the leaf width at all investigated positions in the four connected RILs [4]. According to Hou et al. [11], 40% of the leaf width QTL control the leaf widths in the first leaf above and below the primary ear.

Interestingly, the mQTL appeared to play distinct different effects on adult leaves at different positions. The mQLW7.1 was required for only the first to third leaf widths and mQTL9 affected only the second and third leaf widths. In contrast, meta-QTL mQLW2.2 affected only the sixth and seventh leaf widths. mQLW5 affected L1W to L6W and mQLW1 and mQLW7.2 affected L1W to L5W, which appeared to transition, bridge or switch between mQTL for L1W and L2W and those for L6W and L7W (Table 5). The meta-QTL were composed of loci that jointly affected adjacent leaf widths. This suggests that the architecture of adult leaf widths is mediated by several key common regions and that most of the key genes or loci controlling leaf width might be expressed in a few specific transition leaves (from juvenile to adult leaf transition). Our data suggest that the width of the third and fourth leaf may form a connecting link between the first to second and the sixth to seventh leaves. This possibility is consistent with other studies. Guo et al. (2015) found that the width of the second leaf above the uppermost ear is regulated by more common regions, as compared with the other three leaves, regardless of the germplasm background [4]. Therefore, this leaf might be deemed the transition leaf, which is controlled by more common loci [3]. The width of the fourth leaf shared the highest proportion of loci with the other leaves and might have a transitional effect on other leaves [3]. Both leaf positions in the two studies are similar to the third and fourth leaves in our study. The transcriptomes of the base and tip of developing leaves were different and the expression of genes affecting lignin synthesis was distinct in the leaves of the mature and immature leaves [36].

The QTL involved in leaf width have been found in previous QTL mapping reports [3, 4, 11, 28, 30, 35, 37], although the chromosomal regions for which QTL were located vary across these studies. Base on the marker physical position, we compared the published QTL controlling leaf width with the meta-QTL identified in this study. In the present study, six of the eight meta-QTL were reported to be associated with leaf width in diverse populations across different environments (Table 5). mQLW5 has been confirmed by several studies. Li et al. [28] have detected two leaf width QTL on chromosome 5, one of which is located on 197-202Mb and explains more than 10.2% of the phenotypic variance. Yang et al. [3] found one QTL near umc1852, which contributes 11.8% of the phenotypic variance and controls the first, second and third leaf widths on chromosome 5 between the markers umc1822 and phi048. Ku et al. [35] have also found one important QTL for leaf area on chromosome 5 between bnlg1287 and mmc0282 and have predicted *YABBY15* to be the candidate gene. mQLW5 has also been detected in an enlarged maize association panel [16]. Because the highest correlation occurred between leaf area and leaf width, this region may include a common gene that controls both traits. mQLW2.1, which resides on chromosome 2 between the markers mmc0111 and bnlg2277, is near the *lg1* gene and has been confirmed by Tian et al. [15] in the NAM (Nested Association Mapping) population and further validated by Cai et al. [13]. Yang et al. [3] have found one QTL controlling the width of the sixth and seventh leaves below the tassel. These QTL, which have been detected in different genetic backgrounds and environments, share a high congruence, thus strongly supporting the candidacy of *lg1* for mQLW2.1. The mQLW8.1 located in bin 8.3 has also been identified in a NAM-GWAS study [15]. In addition, one common QTL controlling the leaf width has been mapped to the same region in the RIL derived from B73 × SICAU1212 [3] and Yu82 × D132 [30].

The meta-QTL mQLW7.2 and mQLW9 did not overlap with the reported maize leaf width QTL. Thus, our results identified not only stable and robust QTL validated by other studies but also new QTL, thus further indicating that the genetic architecture of leaf width is complicated and dominated by small but effective alleles. These newly reported QTL not only provide new target genomic regions for further identification and characterization of genes responsible for maize leaf width but also facilitate marker-assisted selection for maize plant architecture improvements to develop hybrids that are better suited to high density planting.

### Application of the QTL associated with leaf widths in maize breeding

Modern maize hybrids provide higher yields than those that were bred several decades ago, primarily because they are adapted to high densities. Plant morphologies that enable efficient light interception at high population densities may increase yield and production [2]. The maize ideotype should exhibit vertically oriented leaves above the ear and horizontally oriented leaves below the ear, such that more light can reach the ear leaves [38]. Furthermore, it is necessary that the appropriate sizes of leaves offer maximized photosynthate without shading the surrounding plant layers [3]. Relatively wider leaves at nodes that are closer to the ear plus narrower leaves that are near the tassel may meet this requirement. However, significant correlations between the different leaf widths at different positions make it difficult to increase the leaf width below the primary ear without increasing the width of the three or four leaves under the tassel. In contrast to the QTL mapping results for leaf widths in different positions, we found a difference in the molecular basis of leaf width among differently positioned leaves. No one QTL that affected all leaf widths was detected. We found two meta-QTL (mQLW7.1 and mQLW9) that affected the widths of the two leaves below the tassel (L1W and L2W) and two meta-QTL that affected the widths of the two leaves below the ear (L6W and L7W). These effects were independent. Our results suggested that the molecular basis of different leaf widths has similarities and differences. Hou et al [11] have also found differences in the molecular basis of leaf width in the leaf above the ear compared with the leaf below the ear. Thus, simultaneously decreasing the width of the two leaves below the tassel (L1W and L2W) and increasing the width of the two leaves (L6W and L7W) near the ear may be possible by manipulating these loci by MAS. The other meta-QTL that have distinct effects on the sizes of leaves at different nodes may be used to alter the plant type further according to the specific application [3]. For example, plants with wider leaves may increase the biomass and taste of forage maize. Therefore, the results of this study may provide valuable information for breeding high density tolerant inbred lines and for using marker-assisted selection for important mQTL.
